# Matrilin-3-Primed Adipose-Derived Mesenchymal Stromal Cell Spheroids Prevent Mesenchymal Stromal-Cell-Derived Chondrocyte Hypertrophy

**DOI:** 10.3390/ijms21238911

**Published:** 2020-11-24

**Authors:** Manjunatha S. Muttigi, Byoung Ju Kim, Bogyu Choi, Inbo Han, Hansoo Park, Soo-Hong Lee

**Affiliations:** 1 School of Integrative Engineering, Chung-Ang University, Seoul 06911, Korea; manjunatha.muttigi@gmail.com; 2 Department of Medical Biotechnology, Dongguk University-Seoul, Seoul 04620, Korea; kbz9861@hanmail.net; 3 Department of Biomedical Science, CHA University, 335 Pangyo-ro, Bundang-gu, 13488 Seongnam, Korea; bgchoi725@gmail.com; 4 Department of Neurosurgery, CHA Bundang Medical Center, CHA University, Seongnam-si 13496, Korea; hanib@cha.ac.kr

**Keywords:** osteoarthritis, hypertrophy, adipose-derived mesenchymal stromal cell, cell spheroids, destabilization of the medial meniscus (DMM)

## Abstract

Adipose-derived mesenchymal stromal cells (Ad-MSCs) are a promising tool for articular cartilage repair and regeneration. However, the terminal hypertrophic differentiation of Ad-MSC-derived cartilage is a critical barrier during hyaline cartilage regeneration. In this study, we investigated the role of matrilin-3 in preventing Ad-MSC-derived chondrocyte hypertrophy in vitro and in an osteoarthritis (OA) destabilization of the medial meniscus (DMM) model. Methacrylated hyaluron (MAHA) (1%) was used to encapsulate and make scaffolds containing Ad-MSCs and matrilin-3. Subsequently, the encapsulated cells in the scaffolds were differentiated in chondrogenic medium (TGF-β, 1–14 days) and thyroid hormone hypertrophic medium (T3, 15–28 days). The presence of matrilin-3 with Ad-MSCs in the MAHA scaffold significantly increased the chondrogenic marker and decreased the hypertrophy marker mRNA and protein expression. Furthermore, matrilin-3 significantly modified the expression of TGF-β2, BMP-2, and BMP-4. Next, we prepared the OA model and transplanted Ad-MSCs primed with matrilin-3, either as a single-cell suspension or in spheroid form. Safranin-O staining and the OA score suggested that the regenerated cartilage morphology in the matrilin-3-primed Ad-MSC spheroids was similar to the positive control. Furthermore, matrilin-3-primed Ad-MSC spheroids prevented subchondral bone sclerosis in the mouse model. Here, we show that matrilin-3 plays a major role in modulating Ad-MSCs’ therapeutic effect on cartilage regeneration and hypertrophy suppression.

## 1. Introduction

Mesenchymal stem cells (MSCs) are thoroughly evaluated cell candidates for cartilage tissue engineering. Previously, the expression of chondrocyte hypertrophy and ossification-associated genes, including collagen type 10, matrix metalloproteinase-13 (MMP-13), alkaline phosphatase, parathyroid hormone-related protein receptor (PTHrP-R), and vascular endothelial growth factor (VEGF), has been reported as a result of in vitro MSC chondrogenic cultures [1,2,3,4,5,6]. The expression of these genes suggests that MSC-derived chondrocytes undergo terminal hypertrophic differentiation involving apoptosis followed by ossification. Additionally, the ectopic implantation of MSC-derived cartilage constructs in vivo resulted in the dedifferentiation of chondrocytes, mineralization, and vascularization [6,7,8]. It is important to note that hypertrophic differentiation is not specific to terminal chondrocyte differentiation and can also happen during osteoarthritis (OA) progression [9,10,11,12,13,14]; hypertrophic differentiation is a major concern in the clinical application of MSCs in hyaline cartilage regeneration because chondrocyte terminal differentiation in the neocartilage may lead to apoptosis and ossification.

Matrilin-3 belongs to a noncollagenous extracellular matrix protein and is functionally considered to be an adaptor protein [15,16]; it is an important component in skeletal development, including mesenchymal differentiation, chondrocyte hypertrophic differentiation, dedifferentiation, and bone mineral density maintenance [17,18,19,20,21,22,23]. Jayasurya et al. showed that matrilin-3 is also essential for TGF-β signaling [24]. As in the case of spondyloepimetaphyseal dysplasia (SEMD) and multiple epiphyseal dysplasias (MEDs), mutations in the matrilin-3 gene cause an aberrant response towards TGF-β and the differentiation of ATDC5 chondroprogenitor cells. Furthermore, wild-type matrilin-3 gene overexpression in ATDC5 chondroprogenitor cells led to spontaneous chondrogenic differentiation, which was confirmed by the increased collagen 2 and aggrecan gene expression and glycosyl amino glycans (GAG) content. Moreover, the mutations of MED and SEMD genes cause an increased expression of collagen 10 resulting in premature hypertrophy [24]. In addition, the role of matrilin-3 in cartilage development, the hypertrophy of chondrocytes, and ossification was confirmed with functional matrilin-3 knockout mice, in which the chondrocytes present in the cartilage prematurely transformed into prehypertrophic and hypertrophic phenotypes, forming an expanded zone of hypertrophy [23]. Moreover, an EGF domain in matrilin-3 binds to BMP2 and acts as an antagonist in order to prevent hypertrophy in chondrocytes. The binding of matrilin-3 to BMP2 prevents BMP-receptor-mediated Smad1 phosphorylation and BMP2-downstream signaling, which are needed for collagen 10 expression in chondrocytes [25].

**Figure 1:**
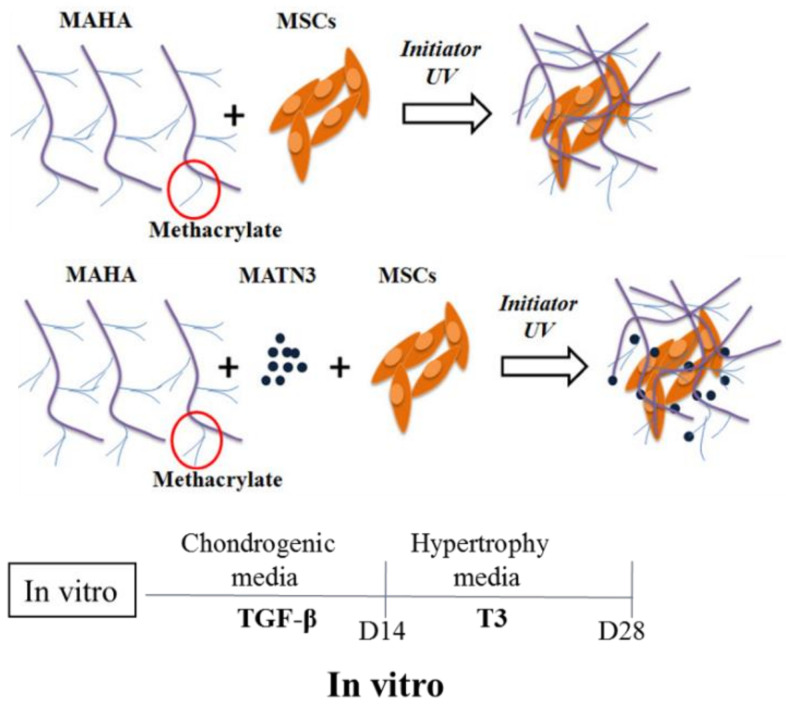
Schematic design of Methacrylated hyaluron (MAHA), Ad-MSC, and matrilin-3 encapsulation; and in vitro chondrogenic and hypertrophic differentiation.

Therefore, we hypothesized that matrilin-3 would suppress Ad-MSC-derived chondrocyte hypertrophy. In this regard, we first induced in vitro chondrogenic differentiation followed by hypertrophic differentiation in a methacrylated hyaluronic acid (MAHA) hydrogel system, and we determined whether the presence of matrilin-3 suppressed hypertrophic differentiation (Figure 1). Next, we assessed the effects of a single-cell suspension or spheroids of matrilin-3-primed Ad-MSCs as a means of obtaining cartilage tissue regeneration and bone mineral density on an in vivo model of destabilization of the medial meniscus (DMM). The morphology of the regenerated cartilage was further assessed using Glasson’s modified semiquantitative osteoarthritis grading system. Our study revealed a favorable effect of matrilin-3 on Ad-MSCs during cartilage regeneration and the suppression of subchondral bone sclerosis.

## 2. Results

### 2.1. Effect of Matrilin-3 Concentration on Ad-MSC Chondrogenesis in MAHA Hydrogel

**Figure 2:**
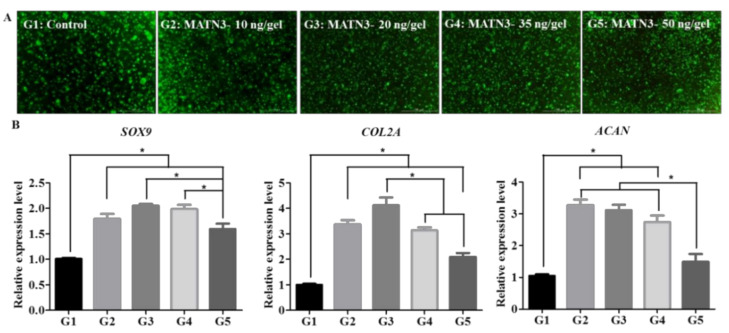
Ad-MSC encapsulation in MAHA hydrogel and dose optimization (14 days). (**A**) Live and dead assay. (**B**) mRNA expression of chondrogenic markers SOX9, collagen 2, and aggrecan. G1: Ad-MSCs; G2: Ad-MSCs + MATN3 (10 ng/gel); G3: Ad-MSCs + MATN3 (20 ng/gel); G4: Ad-MSCs + MATN3 (35 ng/gel); and G5: Ad-MSCs + MATN3 (50 ng/gel). A statistically significant expression is marked with * (*p* < 0.05). Scale bar 200 µm.

Ad-MSCs were encapsulated with matrilin-3 at 10, 20, 35, or 50 ng/gel and induced with a chondrogenic medium for 14 days. Live and dead assays showed that the presence of matrilin-3 did not affect the viability of Ad-MSCs in the MAHA hydrogel (Figure 2A). The presence of matrilin-3 upregulated SOX9, collagen 2, and aggrecan mRNA expression on culture day 14 (Figure 2B). However, matrilin-3 concentrations of 35 ng/gel and 50 ng/gel resulted in a decrease in the expression of SOX9, collagen 2, and aggrecan mRNA on culture day 14 (Figure 2B). These results demonstrate that the regulation of SOX9, collagen 2, and aggrecan expression levels was dependent on the matrilin-3 concentration. Based on the mRNA expression of collagen 2, we selected a matrilin-3 concentration of 20 ng/gel for further experiments.

### 2.2. Effect of Matrilin-3 on Suppression of Ad-MSC-Derived Chondrocyte Hypertrophy

**Figure 3:**
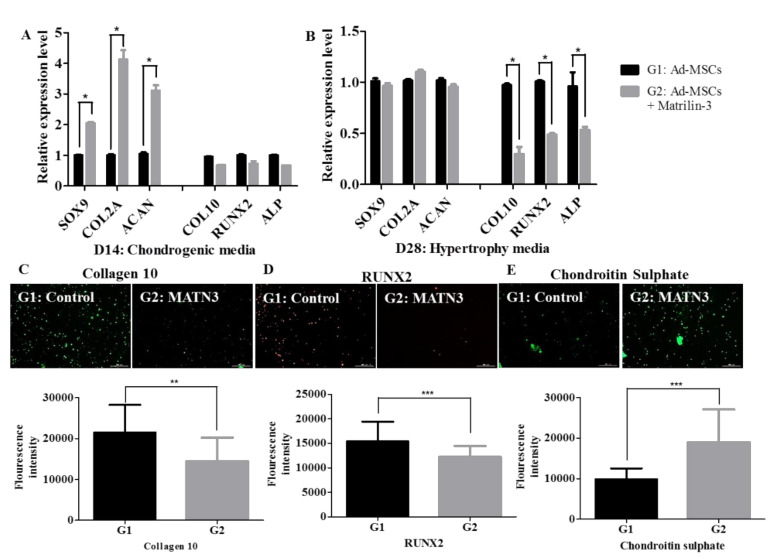
Presence of matrilin-3 suppresses Ad-MSC-derived chondrocyte hypertrophy. (**A**) mRNA expression of chondrogenic, hypertrophy, and ossification markers on day 14. (**B**) mRNA expression of chondrogenic, hypertrophy, and ossification markers on day 28. (**C**–**E**) Immunofluorescence images of hypertrophy, ossification, and chondrogenic markers on day 28. G1: Control, G2: Ad-MSC + matrilin-3. A statistically significant expression is marked with * (*p* < 0.05), ** (*p* < 0.01), and *** (*p* < 0.001). Scale bar 300 µm.

We analyzed the chondrogenic, hypertrophy, and ossification marker expression after the hypertrophy differentiation of Ad-MSC-derived chondrocytes. Ad-MSCs encapsulated with matrilin-3 served as Group 2, and Ad-MSCs encapsulated only in MAHA served as the control (Group 1). Upon chondrogenic differentiation for 14 culture days, the levels of SOX9, collagen 2, and aggrecan mRNA expression were significantly higher in the presence of matrilin-3 (Group 2) when compared with those in Group 1 (Figure 3A). However, we observed no change in the mRNA expression levels of collagen 10, RUNX2, and ALP in both groups on culture day 14 (Figure 3A). After hypertrophy differentiation on culture day 28, the mRNA expression levels for the chondrogenic markers SOX9, collagen 2, and aggrecan were unchanged in both groups (Figure 3B). Conversely, in the presence of matrilin-3 (Group 2), the expression levels of collagen 10, RUNX2, and ALP mRNA were significantly lower when compared with those in Group 1 (Figure 3B). The immunoassay results showed that the collagen 10 (Figure 3C) and RUNX2 (Figure 3D) protein expression levels were significantly decreased in the presence of matrilin-3 (Group 2) when compared with the levels in Group 1. In addition, the chondroitin sulfate (Figure 3E) levels were significantly increased in the presence of matrilin-3 (Group 2) when compared with those in Group 1. These results suggest that matrilin-3 suppressed T3-induced Ad-MSC-derived chondrocyte hypertrophy.

### 2.3. Changes in TGF-β and BMP Expression during Hypertrophic Differentiation

**Figure 4:**
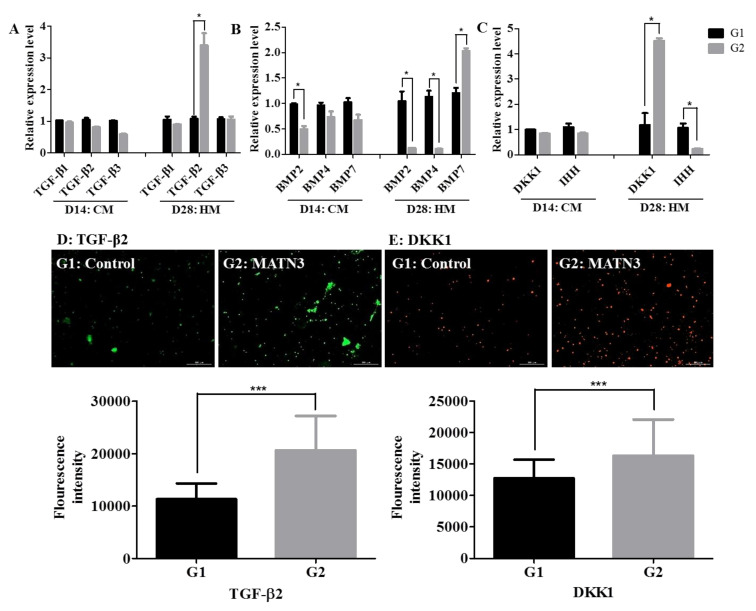
Changes in TGF-β and BMP expression during hypertrophic differentiation. (**A**) mRNA expression of the TGF-β family on days 14 and 28. (**B**) mRNA expression of the BMP family on days 14 and 28. (**C**) mRNA expression of DKK1 and IHH on days 14 and 28. (**D**) Immunofluorescence image of TGF-β2 on day 28. (**E**) Immunofluorescence image of DKK1 on day 28. G1: Control, G2: Ad-MSC + matrilin-3. A statistically significant expression is marked with * (*p* < 0.05), and *** (*p* < 0.001). Scale bar 300 µm.

The levels of TGF-β1, TGF-β2, and TGF-β3 mRNA expression were unchanged after chondrogenic differentiation in both groups (Figure 4A). After hypertrophic differentiation, the mRNA expression of TGF-β2 significantly increased in the presence of matrilin-3 (Group 2) when compared with the control Group 1 (Figure 4A). The BMP2 mRNA expression was significantly decreased after chondrogenic differentiation in the presence of matrilin-3 when compared with the levels in the control group, whereas the BMP4 and BMP7 mRNA expression levels were unchanged (Figure 4B). However, after the hypertrophy differentiation of Ad-MSC-derived chondrocytes, the mRNA expression of BMP2 and BMP4 significantly decreased and that of BMP7 significantly increased in the presence of matrilin-3 (Group 2) when compared with Group 1 (Figure 4B). In addition, the mRNA expression of DKK1 and IHH did not change during chondrogenic differentiation, although the mRNA expression of DKK1 was significantly higher and that of IHH was significantly lower in the presence of matrilin-3 (Group 2) when compared with Group 1 (Figure 4C). The immunofluorescence assay showed that the protein levels of TGF-β2 (Figure 4D) and DKK1 (Figure 4E) significantly increased in the presence of matrilin-3 (Group 2) when compared with Group 1. These results suggest that the presence of matrilin-3 modulated the TGF-β and BMP expression during the hypertrophic differentiation of Ad-MSC-derived chondrocytes.

### 2.4. Matrilin-3 Spheroids Protect Cartilage Degeneration from OA in DMM-Induced Mice

**Figure 5:**
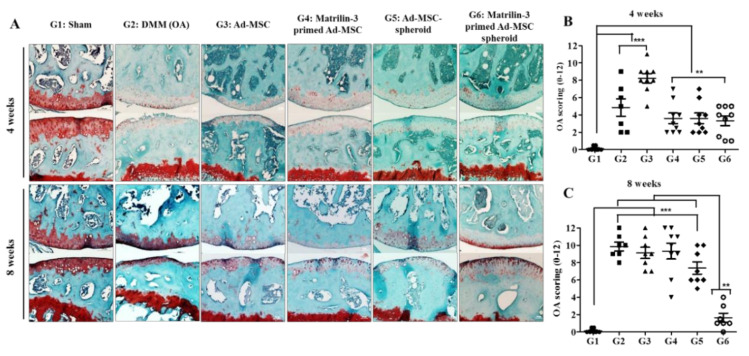
Effect of intra-articular matrilin-3-primed Ad-MSC spheroid treatment on the OA induction of C57Bl/6 mice. Sham, only DMM, Ad-MSC single-cell suspension, matrilin-3-primed Ad-MSC single-cell suspension, Ad-MSC spheroids, and matrilin-3-primed Ad-MSC spheroids were injected into the synovial cavity of OA knee joints and evaluated four and eight weeks after DMM surgery. (**A**) Sagittal histological sections of cartilage were stained with safranin-O (red) and fast green. (**B**,**C**) Quantitative data of OA scoring by Glasson’s modified semiquantitative osteoarthritis grading system (**B**, four weeks and **C**, eight weeks). A statistically significant expression is marked with ** (*p* < 0.01), and *** (*p* < 0.001). Scale bar 100 µm.

To further demonstrate the role of matrilin-3-primed Ad-MSCs, the effects of single-cell suspensions and spheroids were evaluated in a cartilage degeneration by an OA (DMM) environment (Figure 5A). At four and eight weeks after DMM surgery, we observed increased fibrillations extending below the superficial layer, with cartilage erosions extending beyond 80% of its width. Treatment with single-cell Ad-MSCs, matrilin-3-primed single-cell Ad-MSCs, Ad-MSC spheroids, and matrilin-3-primed Ad-MSC spheroids resulted in a roughened articular surface with small fibrillations below the superficial layer at four weeks after surgery. Among all the treated groups, the Ad-MSC single-cell suspension resulted in significantly higher fibrillations at four weeks (***, *p* < 0.001) (Figure 5B). Bone treated with Ad-MSC single-cell and matrilin-3-primed single-cell suspensions showed increased fibrillations extending up to the calcified cartilage, loss of lamina, and cartilage erosion extending from 20% to 80% after eight weeks of surgery (Figure 5C). Treatment with Ad-MSC spheroids resulted in extended fibrillations to the calcified cartilage across less than 20% of the cartilage width after eight weeks of surgery. Interestingly, treatment with matrilin-3-primed Ad-MSC spheroids showed a significant recovery of the cartilage, with no structural lesions, but with a decrease in Safranin-O staining when compared with the other treatments (**, *p* < 0.01) (Figure 5C).

### 2.5. Treatment with Matrilin-3 Spheroids Attenuates Subchondral Bone Sclerosis in a Post-Traumatic OA Model

**Figure 6:**
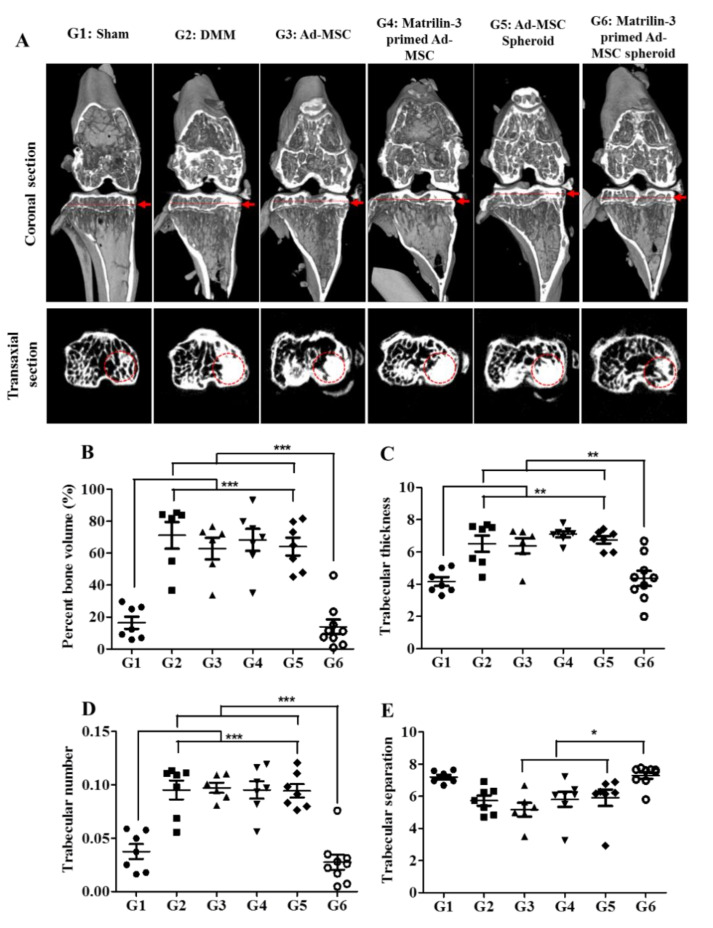
Micro-CT analysis of a 3D-reconstructed subchondral bone layer in an OA joint. (**A**) The red dotted line of each group indicates the location where the epiphysis trabecular bone was cross-sectioned. The cross-sectional image of the ROI (red dotted circle) in the medial subchondral bone compartment was quantitatively analyzed for Skyscan analysis parameters: (**B**) percent bone volume (BV/TV, %), (**C**) trabecular thickness (Tb.Th), (**D**) trabecular number (Tb.N), and (**E**) trabecular separation (Tb.Sp). The graphs show the bone sclerosis of each group evaluated from 15 individual sections of samples as the mean and 95% CI, and a statistically significant expression is marked with * (*p* < 0.05), ** (*p* < 0.01), and *** (*p* < 0.001).

Micro CT was used to analyze changes in the microstructural morphology of the subchondral bone eight weeks after DMM-induced surgery and treatment. As shown in Figure 6A, both coronal and transaxial images showed a higher signal intensity in the DMM, single-cell suspension of Ad-MSCs, matrilin-3-primed single-cell suspension of Ad-MSCs, and Ad-MSC spheroids groups when compared with the matrilin-3-primed Ad-MSC spheroid group (Figure 6A).

Matrilin-3-primed Ad-MSC spheroid-treated groups showed a significantly reduced BV/TV when compared with the groups treated with the Ad-MSC single-cell suspension, matrilin-3-primed Ad-MSC single-cell suspension, and Ad-MSC spheroids (***, *p* < 0.001) (Figure 6B). As shown in Figure 6B,C, BV/TV and Tb.Th were significantly higher (***, *p* < 0.001 and **, *p* < 0.01, respectively) in the DMM mice as well as in the Ad-MSC single-cell suspension, matrilin-3-primed Ad-MSC single-cell suspension, and Ad-MSC spheroid treatment groups when compared with the sham group. However, the Tb.Th was significantly decreased in the matrilin-3-primed Ad-MSC spheroid group when compared with the Ad-MSC single-cell suspension, matrilin-3-primed Ad-MSC single-cell suspension, and Ad-MSC spheroid treatment groups (**, *p* < 0.01) (Figure 6C). Furthermore, the Tb.N was significantly higher in DMM mice (***, *p* < 0.01) as well as in the Ad-MSC single-cell suspension (***, *p* < 0.01), matrilin-3-primed Ad-MSC single-cell suspension (***, *p* < 0.001), and Ad-MSC spheroid (***, *p* < 0.001) treatment groups when compared with the sham group (Figure 6D). Meanwhile, the Tb.N was significantly lower in the group treated with matrilin-3-primed Ad-MSC spheroids than in the groups treated with the Ad-MSC single-cell suspension, matrilin-3-primed Ad-MSC single-cell suspension, and Ad-MSC spheroids (***, *p* < 0.001) (Figure 6D). Furthermore, the Tb.Sp was significantly increased in the matrilin-3-primed Ad-MSC spheroid treatment group when compared with the Ad-MSC single-cell suspension, matrilin-3-primed Ad-MSC single-cell suspension, and Ad-MSC spheroid treatment groups (*, *p* < 0.5) (Figure 6E).

## 3. Discussion

In this study, we demonstrated that Ad-MSC-derived chondrocyte hypertrophy could be suppressed by matrilin-3 in a MAHA hydrogel system. Our initial study showed that matrilin-3 was cytocompatible and did not affect Ad-MSC viability at the tested concentration range (10–50 ng/gel). In addition, the encapsulation of matrilin-3 along with Ad-MSCs increased chondrogenesis. However, it is worth noting that increased matrilin-3 concentrations (35 and 50 ng/gel) reduced the mRNA expression of the chondrogenic marker. These results are consistent with those of our previous study on Ad-MSC pellet culture during chondrogenesis [26]. Therefore, due to its beneficial effects on cartilage regeneration, we selected a concentration of 20 ng/gel for further studies.

Previously, Muller et al. reported that the addition of the T3 hormone, while reducing the dexamethasone concentration and removing TGF-β from the medium, induced MSC hypertrophy in pellet cultures [27]. To mimic these conditions, we first induced the Ad-MSCs encapsulated with or without matrilin-3 in MAHA hydrogels in a chondrogenic medium for 1 to 14 days and in a hypertrophy medium for 15 to 28 days. A quantitative mRNA analysis showed an increase in the chondrogenic marker expression in the presence of matrilin-3 after 14 days of culture in the chondrogenic medium. After the chondrogenic differentiation of Ad-MSCs, we found no change in the mRNA expression of the hypertrophy and ossification markers. These results suggest that matrilin-3 enhances chondrogenic differentiation without altering the basal levels of collagen 10, RUNX2, and ALP mRNA expression. When Ad-MSC-derived chondrocytes were treated with the hypertrophic medium, the expression of chondrogenic markers, such as SOX9, collagen 2, and aggrecan, was unaltered both with and without matrilin-3. However, the mRNA levels of the hypertrophy marker collagen 10 were significantly reduced in the presence of matrilin-3. In addition, the mRNA expression of the ossification markers, such as RUNX2 and ALP, decreased in the presence of matrilin-3 under hypertrophic conditions. At the protein level, there was detectable low-intensity immunostaining for collagen 10 and RUNX2 in the presence of matrilin-3. These findings suggest that Ad-MSC-derived chondrocytes cultured with matrilin-3 under hypertrophic conditions have suppressed hypertrophy and ossification.

The TGF-β and BMP families of genes and proteins are known to be involved in chondrogenic differentiation, terminal hypertrophic differentiation, and ossification [28,29,30,31]. Therefore, the effects of matrilin-3 on the TGF-β and BMP family gene expression were evaluated under chondrogenic and hypertrophic conditions. During chondrogenic differentiation, TGF-β induced mesenchymal condensation and stem cell proliferation, and increased collagen 2 and GAG synthesis. After hypertrophic differentiation, the TGF-β2 mRNA and protein levels increased significantly in the presence of matrilin-3. TGFb-β2 was previously reported to be essential for the maintenance of the chondrocyte phenotype [32], and BMP-2 and BMP-4 are well-known factors in ossification and mineralization [31,33]. The presence of matrilin-3 decreased the BMP-2 and BMP-4 mRNA levels during hypertrophic conditions in our study. Interestingly, the BMP-7 mRNA expression levels were higher in the presence of matrilin-3 under hypertrophic conditions. In addition, the DKK1 mRNA and protein levels increased under hypertrophic conditions in the presence of matrilin-3. BMP-7 acts through the AVCR1 (ALK1) receptor and increases the levels of DKK1 [34,35]. DKK1 is a potent inhibitor of the WNT signaling pathway and thereby inhibits mineralization [34]. Moreover, IHH is known to induce chondrocyte maturation and hypertrophy [36,37], and the presence of matrilin-3 decreased the IHH mRNA expression levels after hypertrophic differentiation. These results suggest that the presence of matrilin-3 modulates the expression of various genes and proteins that suppress hypertrophy during the hypertrophic differentiation of Ad-MSCs derived chondrocytes.

OA is a joint disease that can cause synovium, subchondral plate, and trabecular bone lesions [38]. The cartilage and bone pathologies are involved in reciprocal communication, which may accelerate the progression of post-traumatic osteoarthritis [39,40,41]. Accumulative evidence shows that changes in the remodeling balance between bone and cartilage have an impact on repair and regeneration in OA [41,42]. To confirm the regenerative effects in vivo, matrilin-3-primed Ad-MSCs were injected into the knee joints of the DMM-induced OA model. The matrilin-3-primed Ad-MSC single-cell suspension slowed the progression of cartilage degeneration when compared with the Ad-MSC single-cell suspension alone. Furthermore, the results confirmed that matrilin-3-primed Ad-MSCs, either in a single-cell suspension or spheroid form, effectively prevented the progression of cartilage destruction in the OA model. In addition, we observed that the spheroid form of Ad-MSCs was an ideal treatment candidate for slowing the progression of OA. This could be due to the resemblance of the spheroid to mesenchymal condensation, which is the first stage in the development of cartilage; moreover, MSC spheroids have an increased expression of crucial extracellular matrix (ECM) markers, such as collagen II, collagen IX, aggrecan, tenascin-C, and the cartilage oligomeric matrix [43]. Four weeks after treatment with matrilin-3-primed Ad-MSC spheroids, the cartilage only showed a roughened articular surface, fibrillation extending to the superficial layer, and a loss of lamina, and it recovered with no structural lesions and loss of safranin-O staining at only eight weeks when compared with the other groups.

Changes in the remodeling balance between the bone and cartilage are an important part of the progression of OA. Furthermore, a micro-CT analysis of the percent bone volume, trabecular thickness, trabecular number, and trabecular separation demonstrated the presence of subchondral bone sclerosis in the groups treated with Ad-MSC single-cell suspension, matrilin-3-primed single Ad-MSC, and Ad-MSC spheroids. Interestingly, the percent bone volume and trabecular thickness and number were significantly reduced in the matrilin-3-primed Ad-MSC spheroid-treated group, suggesting that there was a reduction in bone sclerosis when compared with the other treated groups. Notably, matrilin-3 priming and the use of Ad-MSCs in a spheroid formation recovered the cartilage structure and prevented subchondral bone sclerosis. We hypothesized, therefore, that matrilin-3 enhances the regenerative potential of cartilage and possibly inhibits MSC-derived chondrocyte hypertrophy, ossification, and mineralization.

In our study, cartilage damage was prevented by treatments in the following order of increasing potency: Ad-MSC single-cell suspension, matrilin-3-primed Ad-MSC single-cell suspension, Ad-MSC spheroids, and matrilin-3-primed Ad-MSC spheroids. However, we did observe similar effects on subchondral bone sclerosis. Further studies are required to elucidate the effects of the dose and duration of single-cell suspension and spheroids on inflammation-induced bone sclerosis signaling pathways.

## 4. Materials and Methods

### 4.1. Isolation and Culture of Ad-MSCs from Adipose Tissue

Infrapatellar fat pads (adipose tissue) were collected from knee joints during joint-replacement surgery. Informed consent was obtained from all patients, and the tissue harvesting procedure was approved by the institutional ethics committee (IRB number: IACUC150058, CHA University, approval date: 13 May 2015). The Ad-MSCs were isolated from fat tissue as per the previously reported protocol [26].

### 4.2. Preparation of Photopolymerizable Hyaluronic Acid

Hyaluronic acid (500 kDa) was dissolved in distilled water (1% *w*/*v*). Methacrylic anhydride was added to the hyaluronic acid solution to yield MAHA. The mixture was adjusted to pH 8.0–9.0 with NaOH (5 N) for 24 h at 4 °C with constant stirring. Then, the mixture was dialyzed against distilled water for three days (molecular weight cut-off of 50 kDa) (Spectrum Laboratories, CA, USA) to remove unconjugated methacrylic anhydride with fresh water. The MAHA solution was filtered with a 0.2-µm filter and frozen at −80 °C. The frozen MAHA solution was then lyophilized.

### 4.3. Human Ad-MSCs and Matrilin-3 Encapsulation in Hydrogel and Chondrogenesis

Lyophilized MAHA was dissolved in PBS to a 2% (*w*/*v*) aqueous solution. To make the hydrogel, MAHA solution (0.3% final concentration) was mixed with the photoinitiator 0.2% irgacure 2959 (Sigma, Missouri, USA) and Ad-MSCs at a density of 8 × 104 cells per hydrogel. Then, the mixture was put into a polydimethylsiloxane mold with a 5-mm diameter and 2-mm height and irradiated with UV light for 3 min. Along with the Ad-MSCs, matrilin-3 was encapsulated at a concentration of 10 ng/gel (Group 2), 20 ng/mL (Group 3), 35 ng/gel (Group 4), and 50 ng/gel (Group 5). Ad-MSCs only encapsulated in hydrogel were used as control cells (Group 1). These hydrogels were treated in chondrogenic medium containing DMEM-HG, 10% FBS, 100× insulin-transferrin-selenium (ITS), 50 µg/mL ascorbic acid, 100 nM dexamethasone, 1× penicillin and streptomycin, and 10 ng/mL TGF-β. The chondrogenic medium was changed every 48 h. On culture day 14, the cultures were analyzed using live and dead assays and for sex-determining region Y (SRY)-box 9 (SOX9), collagen 2, and aggrecan mRNA expression.

### 4.4. Ad-MSC Chondrogenesis and Hypertrophy Induction

Ad-MSC- and matrilin-3-encapsulated hydrogels were induced by chondrogenic medium for 14 days. The medium was changed to hypertrophy medium containing DMEM-HG, 10% FBS, 100× Insulin-Transferrin-Selenium (ITS), 50 µg/mL ascorbic acid, 1 nM dexamethasone, 1× penicillin and streptomycin, and 1 nM tri-iodothyronine (T3). The hypertrophy medium was supplemented every alternative day for another 14 days. Ad-MSCs with or without matrilin-3 hydrogels were analyzed for SOX9, collagen II, aggrecan, collagen 10, RUNX2, and ALP mRNA expression on culture days 14 and 28. In addition, Ad-MSCs with or without matrilin-3 hydrogels were analyzed for TGF-β1, TGF-β2, TGF-β3, BMP-2, BMP-4, BMP-7, DKK1, and IHH mRNA expression on culture day 28 (Figure 1). Furthermore, an immunofluorescence assay was performed to analyze the protein levels of collagen 10, RUNX2, chondroitin sulfate, TGF-β2, and DKK1.

### 4.5. Live and Dead Assays

A Live/Dead assay (Life Technologies, Seoul, Korea) was performed as per the supplier’s instructions, and images of the cells were captured on day 14 using a Cytation 3 Cell Imaging Multi-Mode Reader (Biotek Instruments, Inc., Winooski, VT, USA).

### 4.6. RNA Extraction and mRNA Expression Analysis

A real-time quantitative polymerase chain reaction (RT-PCR) was performed according to standard procedures. Briefly, the total RNA was extracted using a TRIzol kit (ThermoFisher Scientific, Inc., Waltham, MA, USA). The complementary DNA was subsequently prepared using 0.5 µg of RNA and the primescript RT reagent kit (Takara Bio Inc., Shiga, Japan), and RT-PCR amplification was performed using a StepOnePlus Real Time PCR System. For each target gene, the relative mRNA expression levels were calculated following the 2^−Δ*C*t^ method and using the expression levels of 18S as an internal control. The target primers are listed in Table 1.

### 4.7. Histological Analysis

The hydrogels were fixed in 4% paraformaldehyde for 24 h, embedded in OCT compound, and processed using standard procedures. For the immunofluorescence microscopy, 20-µm-thick histological sections were used, which were fixed for 10 min with 4% paraformaldehyde in PBS at room temperature, washed three times with 1 × PBS, and permeabilized with 0.5% Triton-X for 10 min. Sections were washed with PBS and blocked for 45 min in blocking buffer (5% BSA and 0.5% Tween-20 in 1× PBS) containing 10% normal goat serum at room temperature. For immunostaining, sections were incubated overnight at 4 °C with collagen 10 (1:200, Sigma), RUNX2 (1:200, Abcam, Cambridge, UK), chondroitin sulfate (1:100, Abcam), TGF-β2 (1:200, Abcam), and DKK1 (1:200, Abcam). The secondary antibodies were goat anti-rabbit Alexa Fluor 568 and goat anti-mouse Alexa Fluor 488 (Abcam), which were incubated for 1 h at room temperature. Images were acquired using the Cytation 3 Cell Imaging Multi-Mode Reader (Biotek Instruments, Inc., Winooski, VT, USA). An automated detection of the fluorescent intensities was used to analyze the expression.

### 4.8. Destabilization of Medial Meniscus Osteoarthritis Model

C57BL6 mice were anesthetized with a mixture of tiletamine hydrochloride and zolazepam hydrochloride (Zoletil, 50 mg/kg, Virbac Laboratories, Carros, France) and xylazine (Rompun, 10 mg/kg, Bayer, Seoul, Korea). Animal surgeries were performed according to protocols approved by the CHA University Institutional Animal Care and Use Committee guidelines for the care and use of laboratory animals (Approval number # IACUC160052, approval date: 17 February 2016). The right knee joint capsule medial to the patellar tendon was incised with a number 15 surgical blade, and the joint capsule was opened with a microforcep. Following the fat pad blunt dissection, the medial meniscus (MM) and medial meniscotibial ligament (MMTL) were identified. Following the transaction of the MMTL, the meniscus was free to displace medially, as previously described [44,45]. Mice in the sham group were surgically treated in the same manner, without dissecting the medial meniscus ligament.

### 4.9. Ad-MSC Priming with Matrilin-3, Spheroid Preparation, and Transplantation

Ad-MSCs were seeded at a density of 1500 cells/cm^2^ on cell culture plates and incubated at 37 °C with 5% CO_2_. After 12 h of seeding, the culture medium was changed to serum starvation medium (DMEM-LG and 1× penicillin and streptomycin), and the cells were incubated for 12 h at 37 °C in a CO_2_ incubator. After 12 h of serum starvation, the culture medium was supplemented with 10 ng/mL matrilin-3 that was added every day for five days. Spheroids were prepared using an EZSPHERE 6-well standard plate (well size: 400–500 µm diameter; 100–200 µm depth; 2400 microwells/9.6 cm^2^) (Reprocell Inc., Kanagawa, Japan).

One week after the surgery, matrilin-3-primed Ad-MSCs, whether single-cell suspensions or spheroids, were transplanted to evaluate their effect on cartilage regeneration. The following groups were evaluated: Group 1, sham (*n* = 9); Group 2, DMM (destabilization of the medial meniscus) surgery only (*n* = 9); Group 3, Ad-MSC single-cell suspension (*n* = 9); Group 4, matrilin-3-primed Ad-MSC single-cell suspension (*n* = 9); Group 5, Ad-MSC spheroids (*n* = 9); and Group 6, matrilin-3-primed Ad-MSC spheroids (*n* = 9). For each site, 2 × 10^5^ cells, either single cells or spheroids, were injected with hyaluron solution as the carrier (LG Life Sciences, Seoul, Korea).

### 4.10. Microcomputed Tomography Analysis

The microstructural morphology of the subchondral bone was analyzed using a SkyScan1173 microcomputed tomography (CT) device (Skyscan, Kontich, Belgium) eight weeks after surgery. The X-ray source was set to a voxel size of 50 µm at 90 keV and 88 µA. The exposure time was 500 ms with a frame average of 4. A beam filtration filter made of 1-mm-thick aluminum was used. Data were recorded at every 0.3-degree rotation step for 180 degrees. Image slices were reconstructed using the NRecon software (Skyscan) based on the Fledkamp algorithm and applying the correction for a beam. The subchondral bone of the tibia condyle was chosen as the region of interest (ROI). The outlines of the ROIs were manually drawn using CTAnalyse three-dimensional data analysis software (Skyscan). For these ROIs, the three-dimensional bone volume fraction (BV/TV), trabecular thickness (Tb.Th), bone trabecular number (Tb.N, 1/mm), and bone trabecular separation (Tb.Sp, mm) were calculated [46].

### 4.11. Histological Analysis and Immunohistochemistry

Mice were euthanized at four and eight weeks after surgery. The right knee joints of mice were separated, fixed in 4% paraformaldehyde, and decalcified using 5% nitric acid. Tissue blocks were prepared using paraffin (as described previously) and cut into 4-mm-thick coronal sections, which were stained with Safranin-O/fast green to examine the tissue structure and amount of GAG [47]. The Safranin-O/fast green staining images were used to evaluate OA using Glasson’s modified semiquantitative osteoarthritis grading scale system (Table 2) [44,46].

### 4.12. Statistical Analysis

The results were reported as the mean ± standard error of the mean (SEM), and differences with *p*-values < 0.05 were considered statistically significant. The statistical analysis was performed using a two-tailed unpaired *t*-test, while the analysis of multiple samples was carried out with a one-way analysis of variance (ANOVA) and Tukey’s post-hoc test using SPSS 12.0.1 (SPSS, Inc. Illinois, USA).

## 5. Conclusions

The expression of the hypertrophic phenotype in Ad-MSC-derived chondrocytes is a concern for cartilage tissue regeneration. The in vitro model of Ad-MSC-derived chondrocyte hypertrophy was suppressed in the presence of matrilin-3. The results from the in vivo OA model indicated that matrilin-3-primed Ad-MSC spheroids prevented subchondral bone sclerosis. These results show that matrilin-3 has a significant role in modulating Ad-MSC-mediated cartilage regeneration and hypertrophy suppression.

## Figures and Tables

**Table 1:** Primers used for the quantitative real-time polymerase chain reaction.

Gene	Primer Sequence	Accession Number
18S	5′-GTAACCCGTTGAACCCCATT-3′ 5′-CCATCCAATCGGTAGTAGCG-3′	NR_003286.2
SOX9	5′-GTACCCGCACTTGCACAAC-3′ 5′-TCTCGCTCTCGTTCAGAAGTC-3′	NM_000346.3
Collagen 2a	5′-GGGAGTAATGCAAGGACCA-3′ 5′-ATCATCACCAGGCTTTCCAG-3′	NM_001844.4
Aggrecan	5′-GCCTGCGCTCCAATGACT-3′ 5′-ATGGAACACGATGCCTTTCAC-3′	NM_013227.3
Collagen 10	5′-ACGCTGAACGATACCAAATG-3′ 5′-TGCTATACCTTTACTCTTTATGGTGTA-3′	NM_000493.3
RUNX2	5′-CAGACCAGCAGCACTCCATA-3′ 5′-CAGCGTCAACACCATCATTC-3′	NM_004348
ALP	5′-GACAAGAAGCCCTTCACTGC-3′ 5′-AGACTGCGCCTGGTAGTTGT-3′	NM_000478.4
TGF-β1	5′-ACTACTACGCCAAGGAGGTCAC-3′ 5′-TGCTTGAACTTGTCATAGATTTCG-3′	NM_000660.5
TGF-β2	5′-CAGATGCTTCTGGATTTATGGTATT-3′ 5′-CCAAAGGGTACAATGCCAAC-3′	NM_003238.3
TGF-β3	5′-CGCACACAGCAGTTCTCC-3′ 5′-AAGAAGCGGGCTTTGGAC-3′	NM_003239.3
BMP-2	5′-CGGACTGCGGTCTCCTAA-3′ 5′-GGAAGCAGCAACGCTAGAAG-3′	NM_001200.3
BMP-4	5′-GAGGAAGGAAGATGCGAGAA-3′ 5′-GCACTACGGAATGGCTCCTA-3′	NM_130850.3
BMP-7	5′-TGTCGAGCAGGAAGAGATCC-3′ 5′-ACGCTTCGACAATGAGACG-3′	NM_001719.2
IHH	5′-TGCATTGCTCGTCAAGTC-3′ 5′-CCACTCTCCAGGCGTACCT-3′	NM_002181.3
DKK1	5′-AATGATTTTGATCAGAAGACACACATA-3′ 5′-CAGGCGTGCAAATCTGTCT-3′	NM_012242.2

**Table 2:** Glasson’s modified semiquantitative OA grading system.

Modified Semiquantitative Grading Scale	Score
Normal cartilage	0
Loss of safranin-O with no structural lesions	0.5
Roughened articular surface and small fibrillations	1
Fibrillation below the superficial layer and some loss of lamina	2
Fibrillations extending to the calcified cartilage across less than 20% of the cartilage width	3
Fibrillation and erosions extending from 20 to 80% of the cartilage width	5
Cartilage erosion extending beyond 80% of the cartilage width	6

## Abbreviations

MATN3	Matrilin-3
MAHA	Methacrylated hyaluronic acid
T3	thyroid hormone
TGF-β	transforming growth factor factor-β
BMP	bone morphogenetic protein
DKK1	Dickkopf WNT Signaling Pathway Inhibitor 1
IHH	Indian hedgehog
COL2A	Collagen 2A
ACAN	Aggrecan
